# Lithium attenuates blood–brain barrier damage and brain edema following intracerebral hemorrhage via an endothelial Wnt/β‐catenin signaling‐dependent mechanism in mice

**DOI:** 10.1111/cns.13832

**Published:** 2022-03-28

**Authors:** Dengpan Song, Ya‐Bin Ji, Xiao‐Wen Huang, Yin‐Zhong Ma, Cheng Fang, Lin‐Hui Qiu, Xi‐Xi Tan, Yi‐Man Chen, Sheng‐Nan Wang, Junlei Chang, Fuyou Guo

**Affiliations:** ^1^ Department of Neurosurgery The First Affiliated Hospital of Zhengzhou University Zhengzhou University Zhengzhou China; ^2^ Shenzhen Key Laboratory of Biomimetic Materials and Cellular Immunomodulation Institute of Biomedicine and Biotechnology Shenzhen Institute of Advanced Technology Chinese Academy of Sciences Shenzhen China; ^3^ Department of Neurology Nanfang Hospital Southern Medical University Guangzhou China; ^4^ Department of Neurology Yangjiang People's Hospital Yangjiang China

**Keywords:** blood–brain barrier, intracerebral hemorrhage, lithium, tight junction, Wnt/β‐catenin signaling

## Abstract

**Background:**

Vasogenic cerebral edema resulting from blood–brain barrier (BBB) damage aggravates the devastating consequences of intracerebral hemorrhage (ICH). Although augmentation of endothelial Wnt/β‐catenin signaling substantially alleviates BBB breakdown in animals, no agents based on this mechanism are clinically available. Lithium is a medication used to treat bipolar mood disorders and can upregulate Wnt/β‐catenin signaling.

**Methods:**

We evaluated the protective effect of lithium on the BBB in a mouse model of collagenase IV‐induced ICH. Furthermore, we assessed the effect and dependency of lithium on Wnt/β‐catenin signaling in mice with endothelial deletion of the Wnt7 coactivator *Gpr124*.

**Results:**

Lithium treatment (3 mmol/kg) significantly decreased the hematoma volume (11.15 ± 3.89 mm^3^ vs. 19.97 ± 3.20 mm^3^ in vehicle controls, *p* = 0.0016) and improved the neurological outcomes of mice following ICH. Importantly, lithium significantly increased the BBB integrity, as evidenced by reductions in the levels of brain edema (*p* = 0.0312), Evans blue leakage (*p* = 0.0261), and blood IgG extravasation (*p* = 0.0009) into brain tissue around the hematoma. Mechanistically, lithium upregulated the activity of endothelial Wnt/β‐catenin signaling in mice and increased the levels of tight junction proteins (occludin, claudin‐5 and ZO‐1). Furthermore, the protective effect of lithium on cerebral damage and BBB integrity was abolished in endothelial *Gpr124* knockout mice, suggesting that its protective effect on BBB function was mainly dependent on Gpr124‐mediated endothelial Wnt/β‐catenin signaling.

**Conclusion:**

Our findings indicate that lithium may serve as a therapeutic candidate for treating BBB breakdown and brain edema following ICH.

## Graphical abstract

Lithium significantly decreases hematoma volume and improved neurological outcomes of mice following intracerebral hemorrhage (ICH), and increases blood–brain barrier (BBB) integrity evidenced by reduction of brain edema, Evans blue leakage, and blood IgG extravasation via the Gpr124‐mediated endothelial Wnt/β‐catenin signaling. Lithium may serve as a therapeutic candidate for treating the BBB breakdown and brain edema following ICH.

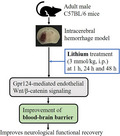

## 1 INTRODUCTION

Intracerebral hemorrhage (ICH) is a common and devastating cerebrovascular disease with high morbidity and mortality that account for 15%–20% of acute strokes.^1^, ^2^, ^3^, ^4^ Despite the large number of patients, effective clinical interventions are lacking.^5^, ^6^, ^7^ In addition, the pathophysiological mechanisms of ICH are complex and unclear. Notably, a series of catastrophic effects caused by the destruction of the blood–brain barrier (BBB) after ICH is one of the main reasons accounting for the poor prognosis of patients.^8^, ^9^ Accumulating evidence has demonstrated that lithium, a clinical drug that is used for the treatment of bipolar mood disorders and can upregulate Wnt/β‐catenin signaling by inhibiting GSK‐3β, exerts neuroprotective effects in experimental ICH models.^10^, ^11^, ^12^, ^13^, ^14^ In contrast, evidence regarding the effect of lithium on the BBB after ICH is very limited. A previous study reported that lithium treatment mitigated damage to the BBB after ICH in rats, with multiple signaling pathways being affected, including the Akt, GSK‐3β, and β‐catenin signaling pathways.^15^ However, whether these effects of lithium on brain injury and BBB function are solely dependent on Wnt/β‐catenin signaling and which cell type plays a major role remain unclear.

The brain endothelium plays an important role in the barrier function of the BBB, and the barrier properties of the brain endothelium are critically regulated by Wnt/β‐catenin signaling.^16^, ^17^ We previously showed that BBB integrity and tolerance were weakened in mice with conditional deletion of endothelial G protein‐coupled receptor 124 (Gpr124), a Wnt7‐specific coactivator of Wnt/β‐catenin signaling, under pathologic conditions, including acute brain ischemia/reperfusion (I/R), and were fully rescued by genetic activation of endothelial‐specific β‐catenin.^18^, ^19^, ^20^ These results indicate that the therapeutic manipulation of BBB integrity via the upregulation of endothelial Wnt/β‐catenin signaling is a potential strategy for combating BBB breakdown in the early stage of ischemic stroke. While lithium has been shown to act as a GSk‐3β inhibitor,^21^ the molecular mechanism underlying its protective effect on the BBB after cerebral hemorrhage has not been well elucidated. In this study, we treated a mouse model of ICH with lithium to elucidate the molecular mechanism underlying its protective effect on the BBB and provided evidence for the clinical use of lithium in the treatment of ICH.

## 2 MATERIALS AND METHODS

### 2.1 Animal protocol and lithium treatment

A total of 120 adult male C57BL/6 mice aged 8–10 weeks and weighing 23 ± 3 g (provided by the Beijing Vital River Laboratory Animal Technologies Co. Ltd) were randomly assigned to four groups (total *n* = 120): (A) a sham‐operated group, in which the mice were subjected to needle insertion only; (B) a sham +lithium chloride (LiCl) group, in which the mice received LiCl after needle insertion (freshly prepared LiCl; #213233, Sigma–Aldrich; 2% in saline injected intraperitoneally (i.p.) at 1 h, 24 h, and 48 h postoperatively); (C) an ICH + LiCl group, in which ICH model animals received LiCl via intraperitoneal injection (intrastriatal bleeding was induced on the right side by stereotactically guided collagenase injection to mimic ICH, and an equal volume of LiCl was administered according the same regimen); and (D) an ICH + vehicle group, in which ICH animals received an equal volume of vehicle (saline) according to the same regimen. The mice were housed at 37 ± 0.5°C on a 12‐h light–dark cycle with free access to water and food. All procedures were approved by the Animal Care and Use Committee of Shenzhen Institute of Advanced Technology, Chinese Academy of Science.

The *Gpr124^flox^* allele was generated previously^18^ and crossed with the tamoxifen‐inducible endothelial driver *Cdh5*‐*CreER* ^22^ on the C57BL/6 background to generate *Gpr124^flox^* ^/^ *^flox^*; *Cdh5*‐*CreER* mice (termed KO mice) and *Gpr124^flox^* ^/+^; *Cdh5*‐*CreER* mice (termed het mice). The Gpr124 het mice did not show any differences from the wild‐type mice. The efficiency and specificity of Gpr124 gene knockout in the brain endothelial cells of the KO mice were confirmed in previous studies and in this study. To induce Gpr124 deletion, 24 KO mice and 24 het mice aged 7–8 weeks were treated with tamoxifen (2 mg/10 g body weight, in corn oil) through an oral feeding needle every other day for 7 days for a total of four doses per mouse. The tamoxifen dosage was based on a previous study.^18^ The mice were allowed to recover from tamoxifen treatment‐related toxicity (washout) for at least 1 week before being subjected to any other surgical procedures or experiments. Similarly, after the ICH model was constructed, an equal volume of LiCl or vehicle control (saline) was administered using the same regimen.

### 2.2 ICH model establishment

The mice were anesthetized with 5% isoflurane and positioned on a stereotaxic frame (Stoelting Co.). A skin incision was made along the sagittal midline to expose the skull. A burr hole was drilled 2.8 mm lateral and 0.3 mm anterior to bregma and 3.8 mm deep, and collagenase VII‐S (sterile‐filtered, 0.5 U in 0.5 μl of sterile saline, Sigma) was then injected slowly into the left basal ganglia at the abovementioned stereotactic coordinates (at a rate of 0.05 μl/min). The needle was left in place for an additional 10 min and withdrawn slowly (at a rate of 1 mm/min) to prevent reflux. The burr hole was sealed with bone wax, the skin was sutured, and the mice were allowed to recover under observation. For the mice in the control group, holes were drilled in the skull at the same location, but collagenase was not injected. Mice that died before the end of the study were excluded.

### 2.3 Neurological score evaluation

Neurological deficit scores were assigned, and the grab test and horizontal ladder test were performed at 72 h after ICH by a blind observer as previously described.^19^ Animals were excluded if no neurological deficits were observed after ICH according to the pre‐established exclusion plan.

### 2.4 Hematoma volumetric analysis

Mice were euthanized under deep isoflurane anesthesia, and 2‐mm‐thick coronal brain slices were prepared using a mouse brain matrix. The brain slices were scanned, and the hematoma size was calculated by a blinded investigator using ImageJ software (NIH). A binary image in which the hematoma appeared red and the remaining brain tissue appeared white was created. The total hematoma area was converted to a volumetric measurement by multiplying it by the slice thickness and is expressed as the mean hematoma volume (mm^3^).

### 2.5 Brain water content examination

The mice were euthanized under deep isoflurane anesthesia 3 days after ICH induction, and their brains were removed quickly. The ipsilateral and contralateral hemispheres of the brains were dissected and weighed with a precise electronic balance (model AE 100; Mettler Instrument Corp.) immediately after harvesting to determine the wet weights. The brain samples were then dried at 100°C for 24 h to determine the dry weight and brain water content, which was calculated as [(wet weight − dry weight)/wet weight] × 100%.

### 2.6 Measurement of Evans blue (EB) leakage

An EB extravasation assay was performed to assess the permeability of the BBB after ICH according to previous reports.^19^ The results are shown as the ratio of EB content in the ipsilateral (ipsi) hemisphere to that in the contralateral (contra) hemisphere.

### 2.7 Western blot analyses

After the mice were deeply anesthetized, their brains were removed, and 1 mm of brain tissue around the hematoma was collected. Protein extraction and WB analysis of brain tissue were performed according to previous reports.^19^ The membranes were probed with primary rabbit anti‐claudin‐5 (1:1000, #34‐1600, Thermal Fisher), rabbit anti‐ZO‐1 (1:1000, #40‐2200, Thermal Fisher), rabbit anti‐occludin (1:1000, #71‐1500, Thermal Fisher), rabbit anti‐laminin (1:1000, #PA5‐115490, Thermal Fisher), rabbit anti‐collagen IV (1:1000, #PA1‐28534, Thermal Fisher), and mouse anti‐β‐actin (1:1000, #66009‐1, ProteinTech) antibodies at 4°C overnight. The relative intensity of each protein signal was quantified via densitometric analysis using ImageJ software.

### 2.8 Immunohistochemistry staining

Immunohistochemistry staining was performed as described previously.^19^ Three slices from the brain of each mouse were stained, and three images of each slice were acquired randomly. Therefore, nine images were acquired per mouse and averaged for quantification. The antibodies used included hamster anti‐mouse CD31 (1:200, #MAB1398Z, Millipore), donkey anti‐mouse IgG (1:500, #715‐545‐150, Jackson ImmunoResearch), rabbit anti‐claudin‐5 (1:40, #34‐1600, Thermal Fisher), rabbit anti‐ZO‐1 (1:100, #40‐2200, Thermal Fisher), rabbit anti‐laminin (1:200, #L9393, Sigma–Aldrich), and rabbit anti‐active β‐catenin (1:50, #8814S, CST) antibodies. The images were processed and analyzed using ImageJ software.

### 2.9 Statistical analysis

Statistical analysis was performed with the SPSS 20.0 for Windows software package (SPSS, US). The data are expressed as the mean ± standard errors (SEs). The Shapiro–Wilk test was used to assess the normality of the data distribution. Statistical differences among multiple groups were analyzed by one‐way analysis of variance (ANOVA) followed by Tukey's multiple comparisons test, while those between two groups were analyzed by unpaired t tests. Statistical significance was defined as *p* < 0.05.

## 3 RESULTS

### 3.1 Lithium treatment alleviated neurological damage in adult mice following acute hemorrhagic stroke

**FIGURE 1:**
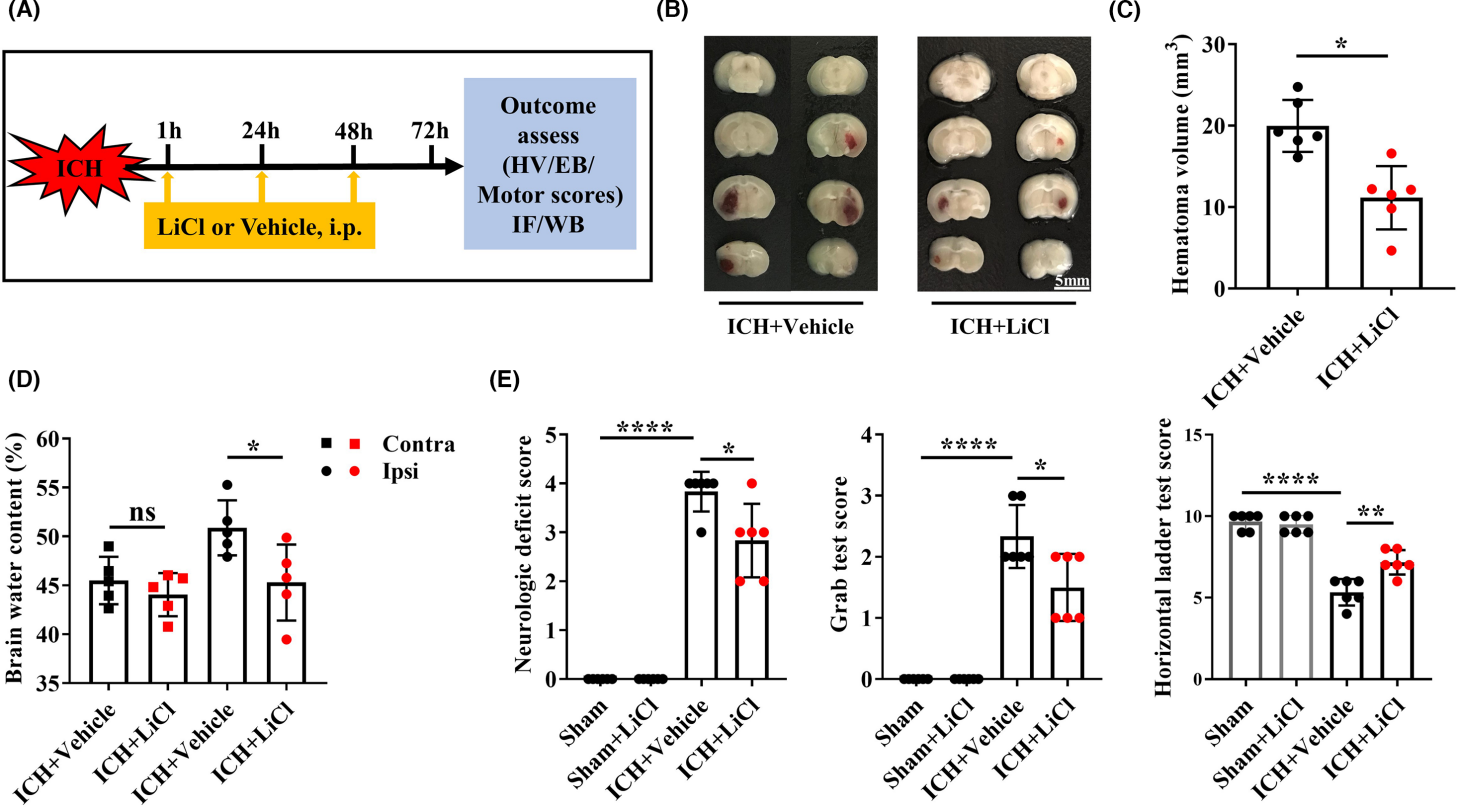
Lithium improved stroke outcomes in adult mice with ICH. (A) Experimental procedure. (B) Hematoma volumes in mice with ICH after 3 mmol/kg LiCl or vehicle treatment. (C) Quantification of the hematoma volumes in mice. *n* = 6 mice per group. (D) Brain water content in the contralateral hemisphere (Contra) and ipsilateral hemisphere (Ipsi). *n* = 5 mice per group. (E) The neurologic deficit scores as well as the grab test and horizontal ladder test performances of mice with ICH treated with LiCl or vehicle. *n* = 6 mice per group. *p < 0.05. ICH, intracerebral hemorrhage; Contra, contralateral hemisphere; Ipsi, ipsilateral hemisphere

Red hematoma tissues were detected in brain sections harvested from adult mice subjected to ICH, and the hematoma volume was reduced in the group administered three intraperitoneal injections of LiCl at a dose of 3 mmol/kg (11.15 ± 3.89 mm^3^) compared with the group administered the vehicle control (19.97 ± 3.20 mm^3^; *p* = 0.0016; Figure 1A–C). Brain water content measurements revealed that LiCl reduced cerebral edema in the ipsilateral hemisphere without affecting the contralateral hemisphere (*p* = 0.0312; Figure 1D). LiCl treatment reduced neurological deficit scores (*p* = 0.0169) and improved grabbing (*p* = 0.0219) and horizontal ladder (*p* = 0.0023) movements (Figure 1E).

### 3.2 Lithium protected BBB function in adult mice following acute hemorrhagic stroke

**FIGURE 2:**
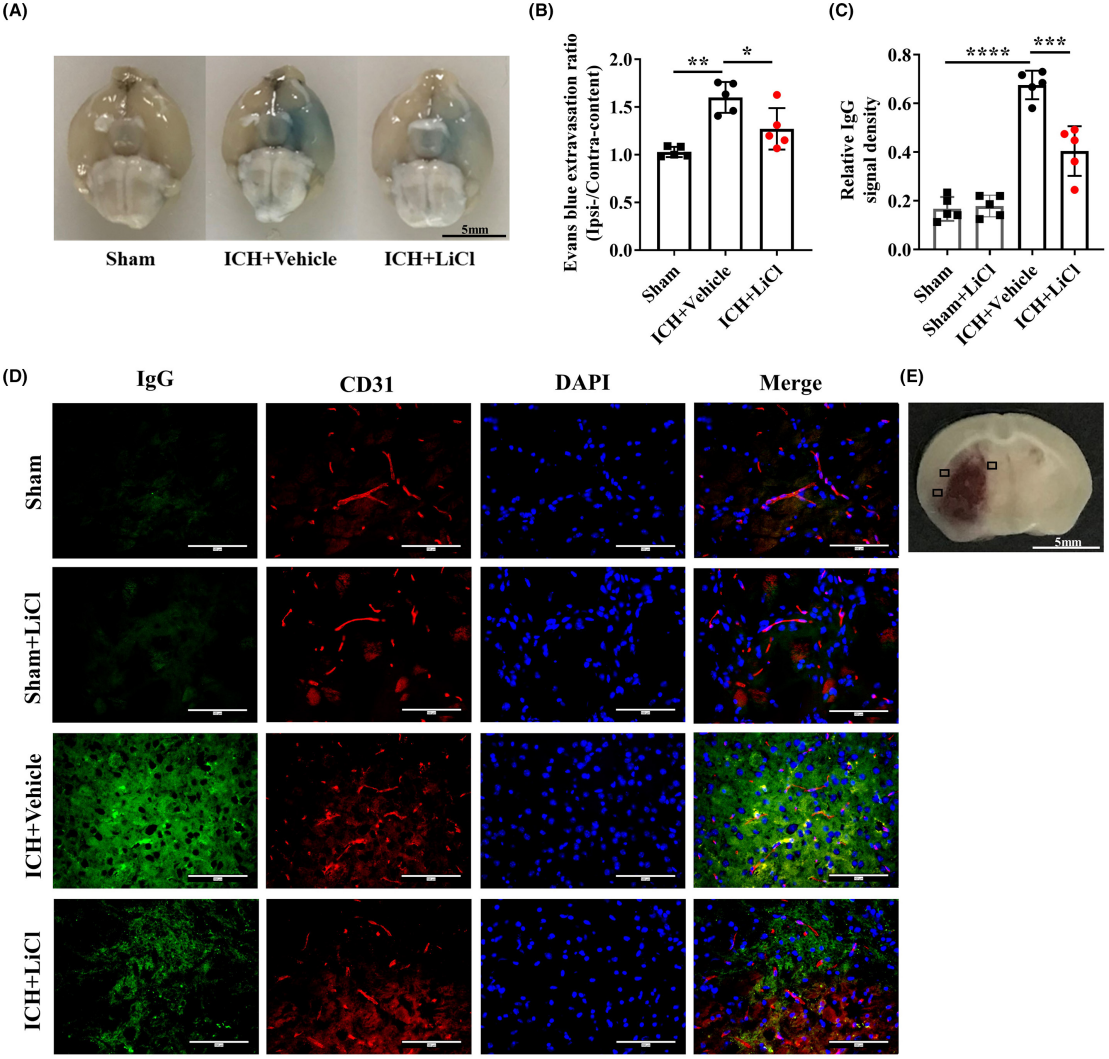
Lithium protected the BBB in adult mice with ICH. (A) Representative images of the brain showing EB leakage in control mice with ICH and reduced leakage in mice treated with LiCl at a dose of 3.0 mmol/kg. (B) Quantitation of EB fluorescence. *n* = 5 mice per group. (C) Quantification of relative IgG signal densities. *n* = 5 mice per group. (D) Immunofluorescence staining of endogenous plasma IgG (green) leakage from vessels (red) after ICH in mice treated with or without LiCl. (E) Schematic illustration showing where the images in (D) were taken from. Perihematomal regions (indicated by black squares) were selected and imaged. Scale bar, 100 μm. *p < 0.05, **p < 0.01. ICH, intracerebral hemorrhage; BBB, blood–brain barrier

We determined the severity of BBB breakdown in the hemorrhagic hemisphere via an EB extravasation assay.^23^ EB leakage was significantly reduced in the mice treated with LiCl at a dose of 3.0 mmol/kg compared with the mice treated with the vehicle control (*p* = 0.026; Figure 2A,B). BBB leakage was additionally assessed by measuring the IgG extravasation from vessels around the hematoma areas into the mouse brain parenchyma. IgG leakage into the blood was reduced in mice treated with LiCl compared with mice treated with the vehicle control (*p* = 0.0009; Figure 2C–E). These data indicate that LiCl treatment significantly improved BBB function after acute hemorrhagic stroke.

### 3.3 Lithium preserved BBB function by regulating endothelial tight junctions following acute hemorrhagic stroke

**FIGURE 3:**
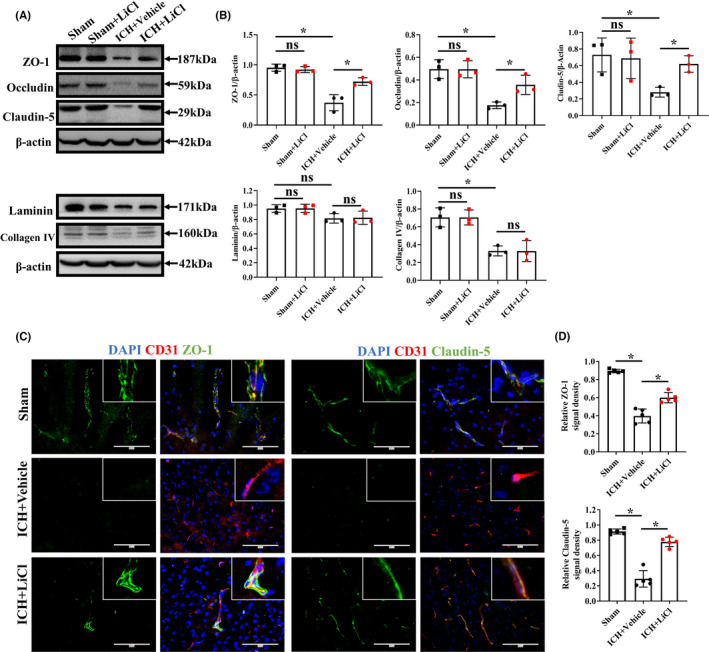
Lithium protected the BBB by regulating endothelial tight junctions following ICH. (A) Levels of tight junction protein markers (ZO‐1, occludin and claudin‐5) and basement membrane proteins in ICH mice as measured by Western blotting. (B) Quantitation of the bands in (A). *n* = 3 mice per group. The experiments were repeated twice, and representative results are shown. (C) Coimmunofluorescence staining for claudin‐5 or ZO‐1 (green) and CD31 (red) around the hematoma areas. Scale bar, 100 μm. (D) The positive signal density was normalized to the CD31 signal area. *n* = 5 mice per group. Scale bar, 100 μm. *p < 0.05, **p < 0.01. ICH, intracerebral hemorrhage

To observe the improvement in BBB integrity induced by lithium treatment after acute hemorrhagic stroke, we evaluated the major components of endothelial tight junctions. We measured the levels of more marker proteins than a previous study.^24^ LiCl had no apparent effects on the expression levels of marker proteins, as determined by Western blotting (Figure 3A,B). After ICH, LiCl treatment significantly reversed the reduction in and largely maintained the protein levels of ZO‐1 (*p* = 0.0147), occludin (*p* = 0.0259), and claudin‐5 (*p* = 0.0069). Consistent with this finding, the coverage of ZO‐1 (*p* = 0.0015) and claudin‐5 (*p* < 0.0001) on vessels around the hematoma areas was greater in mice treated with LiCl than in control mice as determined by immunofluorescence staining (Figure 3C,D). Furthermore, we assessed the expression of basement membrane components by Western blot analysis. ICH and LiCl treatment did not significantly increase the expression of laminin (*p* = 0.9084) or collagen IV (*p* = 0.9702; Figure 3A,B). Taken together, our results show that lithium does not comprehensively protect the components of the BBB except for endothelial tight junctions after hemorrhagic stroke.

### 3.4 Lithium upregulated endothelial Wnt/β‐catenin signaling after acute hemorrhagic stroke

**FIGURE 4:**
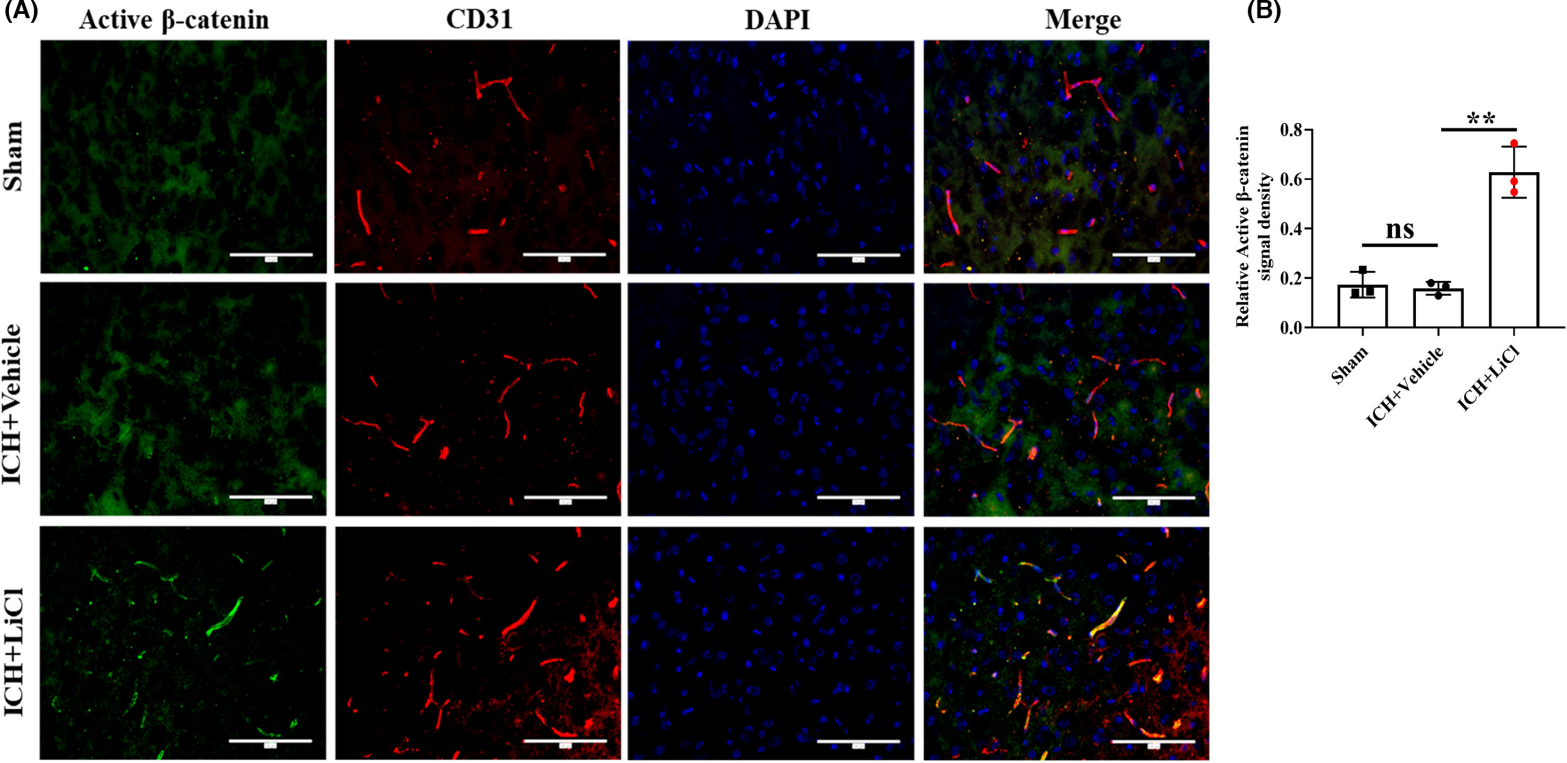
Lithium upregulated Wnt/β‐catenin signaling in the brain endothelium after ICH in vivo. (A) Coimmunofluorescence staining for active β‐catenin (green) and CD31 (red) around the hematoma areas. Scale bar, 100 μm. (B) The active β‐catenin signal density was normalized to the CD31 signal area. *n* = 3 mice per group. **p < 0.01. ICH, intracerebral hemorrhage

Endothelial cells are the main components of the BBB, and the importance of the Wnt/β‐catenin signaling pathway under both physiological and pathological conditions has been reported in many studies.^25^, ^26^ The Wnt/β‐catenin signaling pathway has been shown to ameliorate cerebral and neurological deficits caused by the regulated expression of multiple downstream target genes.^27^, ^28^ Lithium has been shown to definitively upregulate Wnt/β‐catenin signaling, but its effect on ICH‐induced endothelial injury via this mechanism is unclear. Hence, we verified that lithium protects the BBB by upregulating endothelial Wnt/β‐catenin signaling after ICH. In vivo, we found that the endothelial expression of active β‐catenin around the hematoma area, as assessed by immunofluorescence staining, was significantly increased in mice treated with LiCl compared with control mice (*p* = 0.0016; Figure 4A,B). Taken together, our in vivo results indicate that lithium mitigated cerebral ICH injury‐induced BBB disruption by upregulating endothelial Wnt/β‐catenin signaling.

### 3.5 The protective effects of lithium on the BBB were mainly dependent on Gpr124‐mediated endothelial Wnt/β‐catenin signaling

**FIGURE 5:**
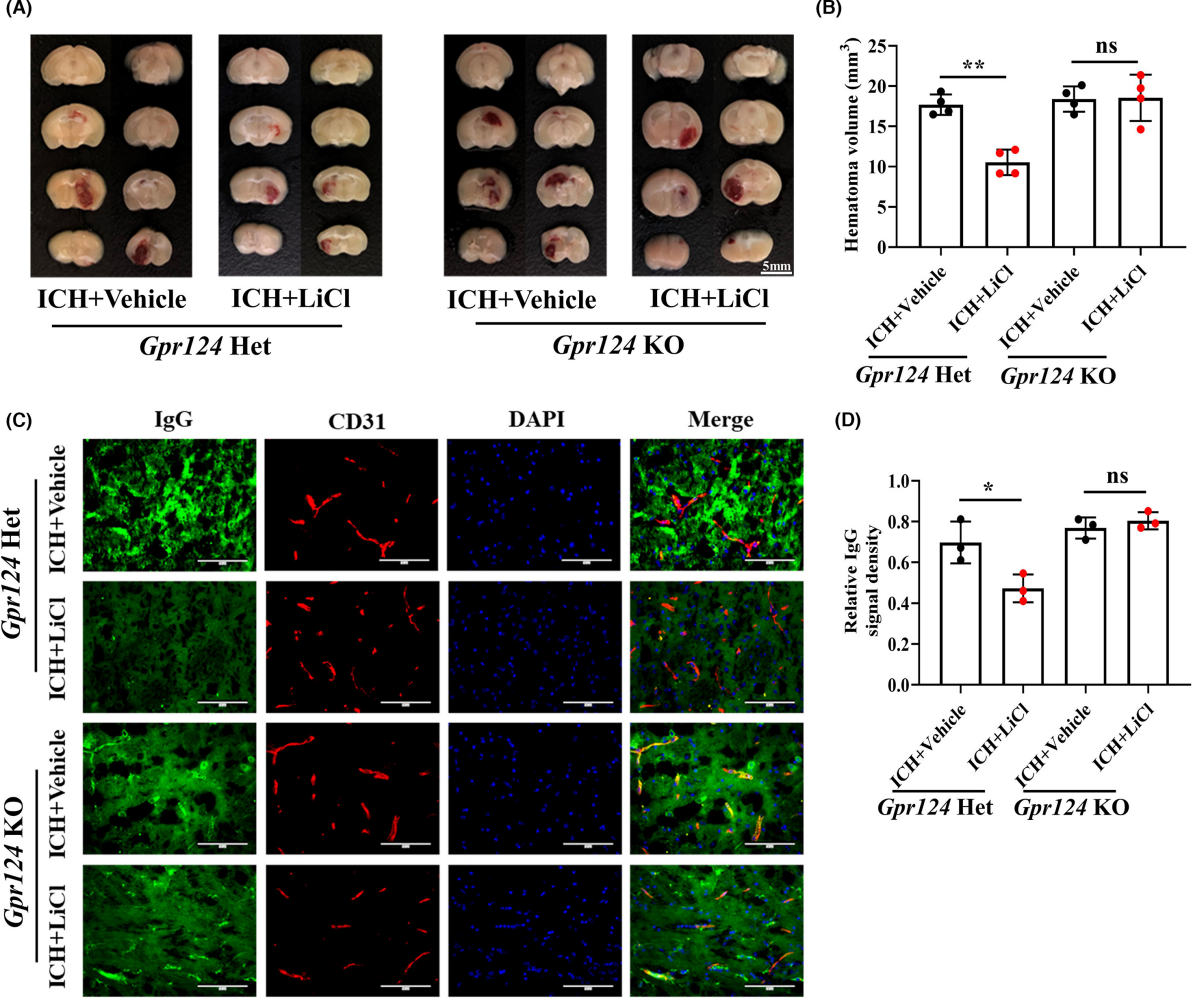
Lithium improved outcomes after ICH through Gpr124‐mediated endothelial Wnt/β‐catenin signaling. (A) Hematoma volumes in mice with ICH. (B) Quantification of the hematoma volumes in mice. *N* = 4 mice per group. (C) Immunofluorescence staining of endogenous plasma IgG (green) leaking from vessels (red) after ICH in mice treated with or without LiCl. Scale bar, 100 μm. (D) Quantification of the relative IgG signal densities. n = 3 mice per group. *p < 0.05, **p < 0.01. ICH, intracerebral hemorrhage; BBB, blood–brain barrier

*Gpr124* knockout induces deficiency of endothelial Wnt/β‐catenin signaling.^18^ To determine whether the protective effect of lithium on the BBB depends on the upregulation of endothelial Wnt/β‐catenin signaling, we generated *Gpr124^flox^* ^/^ *^flox^*; *Cdh5*‐*CreER* (KO) mice and *Gpr124^flox^* ^/+^; *Cdh5*‐*CreER* (het) mice based on previous research.^19^ While the hematoma volume was significantly reduced in het mice compared with vehicle‐treated control mice (*p* = 0.0004), it was not reduced in KO mice compared with vehicle‐treated control mice (*p* = 0.9230; Figure 5A,B). Consistently, IgG extravasation from vessels around the hematoma areas was observed, but no apparent changes were observed in KO mice after LiCl treatment (*p* = 0.4072; Figure 5C,D). In summary, these data demonstrate that the protective effect of lithium on the BBB is mainly dependent on Gpr124‐mediated endothelial Wnt/β‐catenin signaling.

## 4 DISCUSSION

Drugs and surgery are required in combination for the treatment of patients with acute cerebral hemorrhage.^29^, ^30^ In addition to symptomatic treatment, protecting the BBB in the early stage of ICH has great clinical value. Unfortunately, no suitable drugs are available to protect the BBB. As a clinical drug that has been used for a long time, lithium can potentially be used for the treatment of cerebral hemorrhage. Previous studies have found that lithium plays a role in protecting the brain after cerebral hemorrhage, specifically affecting apoptosis, inflammation, and autophagy.^10^, ^31^, ^32^ However, the underlying molecular mechanisms by which lithium protects against BBB damage following ICH remain unclear. Here, we utilized an ICH model to investigate the protective effects of lithium on brain damage and determine the mechanisms underlying the protective effect of lithium on the BBB.

Disruption of BBB integrity and the subsequent increase in permeability are the major aspects of brain injury after ICH.^33^, ^34^ BBB damage causes brain edema^35^ and enlarges the hematoma, increasing intracranial pressure and possibly resulting in a shift in the midline.^36^, ^37^ Previous studies have found that edema peaks on the third day after ICH,^38^ further indicating that BBB integrity is poor at this time point. Therefore, we evaluated the alleviating effect of lithium on BBB damage after ICH. Here, we found that lithium treatment significantly attenuated BBB breakdown after ICH using EB and blood IgG leakage assays.

The canonical Wnt/β‐catenin signaling pathway is a key regulator of the BBB and is critical for the stabilization of brain endothelial tight junctions.^39^, ^40^, ^41^ Lithium, the first GSK‐3β inhibitor, increases the phosphorylation of GSK‐3β at Ser9 in vivo and upregulates Wnt/β‐catenin signaling.^42^ Although a previous study reported the effect of lithium on BBB damage in experimental ICH, the mechanism was not clarified.^15^ Whether the protective effects of lithium on BBB function are solely dependent on Wnt/β‐catenin signaling and which cell type (endothelial, neuronal or glial cells) mediates the effect remain unknown, as the previous study used whole brain tissue lysate for protein measurements. We herein revealed that the Wnt/β‐catenin signaling in endothelial cells is essential for the protective effect of lithium on the BBB, as shown by both an endothelial‐specific Wnt signaling deficiency mouse model and upregulation of active β‐catenin levels by lithium in brain endothelial cells.

Activating the Wnt signaling pathway may have some potential risks, especially in tumor‐related diseases. For example, in many kinds of tumors, cancer cell survival, growth, and differentiation depend on Wnt/β‐catenin signaling.^43^ Upregulation of Wnt/β‐catenin might thus increase the risk of tumor generation or growth. It has been reported that Wnt/β‐catenin signaling plays an important role in prostate cancer and glioma, promoting self‐renewal or expansion.^44^, ^45^ In contrast, melanoma growth is slowed by the activation of Wnt/β‐catenin signaling.^46^ It is worth noting that although the role of Wnt/β‐catenin signaling in tumor development is still controversial, tumor progression is a long‐term process, in contrast to the acute onset of cerebral hemorrhage. In addition, Wnt/β‐catenin signaling also plays an important role in vascular repair after traumatic brain injury.^47^ In the model used herein, disruption of the integrity and permeability of the BBB occurred at an early stage, which was consistent with a previous study.^48^, ^49^ Thus, we believe that the benefits of Wnt/β‐catenin signaling activation in the short term after ICH outweigh the potential side effects. Indeed, in our previous study, we also inhibited the expression of DKK‐1 to promote the activation of Wnt/β‐catenin signaling and thereby treat BBB damage after acute cerebral hemorrhage in rodents.^38^ However, as a clinical drug, lithium has a very large advantage over other activators, as it has a clear pharmacological basis and recognized neuroprotective effect and can be directly applied clinically to benefit ICH patients. We did not observe obvious expression of active β‐catenin in the brains of mice in the sham operation and ICH groups. It is possible that under normal conditions, a low level of active β‐catenin is sufficient to maintain the physiological functions of the endothelium, but in pathologic states such as ICH, a higher level of active β‐catenin is required. In fact, our results showed that the expression level of active β‐catenin around the hematoma tissue was significantly increased after lithium treatment.

Tight junction proteins in brain endothelial cells play a vital role in the integrity and permeability of the BBB.^50^, ^51^, ^52^ We previously revealed that the activation of the Wnt/β‐catenin signaling pathway by DKK‐1 knockdown upregulates the expression of the tight junction protein ZO‐1 after cerebral hemorrhage.^12^ Here, we found that lithium treatment rescued the ICH‐induced reduction in claudin‐5, occludin, and ZO‐1 expression in the endothelium but detected no obvious changes in sham mice after lithium treatment. We measured tight junction protein levels and observed upregulation only under pathological conditions (in the ICH group) after lithium treatment, which also indicated that the activation of the Wnt/β‐catenin signaling pathway rescued the structure of the BBB under pathological conditions.

GPR124 is an orphan receptor in the adhesion family of G protein‐coupled receptors (GPCRs). Studies have found that GPR124 acts as a WNT7A/7B‐specific synergistic activator via an unclear direct or indirect mechanism and plays a role in canonical Wnt/β‐catenin signaling.^18^ GPR124 regulates Wnt signal transduction in endothelial cells under the pathological state of stroke in adult mice, thereby regulating the integrity of the BBB.^18^ This finding demonstrates the potential of GPR124 as a therapeutic target for human central nervous system diseases involving BBB destruction. In this study, we clearly demonstrated that lithium attenuates BBB injury after intracerebral hemorrhage. To the best of our knowledge, this report is the first to demonstrate that lithium improves BBB injury and brain edema after intracerebral hemorrhage through endothelial GPR124‐mediated Wnt/β‐catenin signaling. These findings may also indicate that the activation of Wnt/β‐catenin signaling by lithium is closely related to GPR124.

## 5 CONCLUSION

In summary, our study reveals that lithium ameliorates cerebral edema and improves outcomes by activating endothelial Wnt/β‐catenin signaling to protect tight junction proteins of the BBB and provides a potential treatment option for ICH in the early stage.

## CONFLICT OF INTEREST

The authors declare no conflict of interest.

## AUTHOR CONTRIBUTIONS

Fuyou Guo and Junlei Chang designed and supervised the study. Dengpan Song, Ya‐Bin Ji, Xiao‐Wen Huang, Yin‐Zhong Ma, and Cheng Fang performed the experiments and data analyses. Lin‐Hui Qiu, Xi‐Xi Tan, Yi‐Man Chen and Sheng‐Nan Wang performed basic studies. Dengpan Song and Ya‐Bin Ji wrote the manuscript, and Junlei Chang and Fuyou Guo revised the manuscript.

## Supporting information

Supplementary Material

## Data Availability

The data that support the findings of this study are available from the corresponding author upon reasonable request.
